# Does the Micronutrient Molybdenum Have a Role in Gestational Complications and Placental Health?

**DOI:** 10.3390/nu15153348

**Published:** 2023-07-27

**Authors:** Vladimira Foteva, Joshua J. Fisher, Yixue Qiao, Roger Smith

**Affiliations:** 1 Mothers and Babies Research Program, Hunter Medical Research Institute, Newcastle, NSW 2305, Australia; joshua.fisher@newcastle.edu.au (J.J.F.); roger.smith@newcastle.edu.au (R.S.); 2 School of Medicine and Public Health, University of Newcastle, Newcastle, NSW 2308, Australia; 3 Academy of Pharmacy, Xi’an Jiaotong Liverpool University, Suzhou 215000, China; yixue.qiao@uon.edu.au

**Keywords:** molybdenum, micronutrients, pregnancy, molybdoenzymes, oxidative stress, gestational diabetes mellitus, antioxidants

## Abstract

Molybdenum is an essential trace element for human health and survival, with molybdenum-containing enzymes catalysing multiple reactions in the metabolism of purines, aldehydes, and sulfur-containing amino acids. Recommended daily intakes vary globally, with molybdenum primarily sourced through the diet, and supplementation is not common. Although the benefits of molybdenum as an anti-diabetic and antioxidant inducer have been reported in the literature, there are conflicting data on the benefits of molybdenum for chronic diseases. Overexposure and deficiency can result in adverse health outcomes and mortality, although physiological doses remain largely unexplored in relation to human health. The lack of knowledge surrounding molybdenum intake and the role it plays in physiology is compounded during pregnancy. As pregnancy progresses, micronutrient demand increases, and diet is an established factor in programming gestational outcomes and maternal health. This review summarises the current literature concerning varied recommendations on molybdenum intake, the role of molybdenum and molybdoenzymes in physiology, and the contribution these play in gestational outcomes.

## 1. Introduction

Pregnancy is a complex biological process, necessitating physiological and anatomical changes to create a maternal environment conducive to nurturing the growing foetus [1]. The maternal environment during the gestational period is a modulator of lifelong health outcomes for the offspring. Suboptimal foetal circumstances are associated with increased rates of mortality in neonates, and the early onset of coronary heart disease, hypertension, and renal and metabolic disorders in adulthood [2,3,4]. In addition, suboptimal maternal and in utero environments have been shown to precede gestational complications including foetal growth restriction (FGR), gestational diabetes (GDM), and pre-eclampsia [5,6,7]. These gestational complications are frequently associated with oxidative stress and inflammation, resulting in inadequate placental function due to damage from reactive oxygen species. Central to these processes is maternal nutrition during gestation [8,9]. Micronutrients, essential elements required in trace quantities and sourced from the diet, are integral to all metabolic processes including cell signalling, differentiation and proliferation, and critically, fundamental for antioxidant function [10]. Deficient micronutrient status is common both in low- and middle-income countries with seasonal diets and chronic undernutrition, as well as in high-income countries with diets characterised by calorie-rich but nutrient-poor food, which is high in fat, sugars, and sodium, but low in vitamin and mineral content. Therefore, micronutrient supplementation has been suggested as a potential modifiable, low-risk treatment for improving maternal-foetal health outcomes globally [11]. Furthermore, placental and umbilical cord tissue is increasingly used as a non-invasive exposure biomarker for element biomonitoring studies, and may reflect maternal micronutrient status [12,13,14].

Of the 14 essential minerals necessary for human health and gestation, which encompass calcium, chromium, copper, fluoride, iodine, iron, magnesium, manganese, molybdenum, phosphorous, potassium, selenium, sodium, and zinc at a range of dietary intakes [15], the micronutrient molybdenum (Mo) has received little attention. Molybdenum belongs to the transition metal group of the periodic table, and forms the active site of five key enzymes in humans, termed molybdoenzymes. Molybdoenzymes metabolise a wide range of endogenous and exogenous compounds, including sulfur-containing amino acids by the molybdoenzyme sulfite oxidase, purines by the molybdoenzyme xanthine oxidoreductase, aldehydes by the molybdoenzyme aldehyde oxidase and amidoximes by the mitochondrial amidoxime reducing components [16]. An inability to synthesise the molybdoenzyme sulfite oxidase is lethal, due to accumulation of sulfite, but the main endogenous substrates for other molybdoenzymes, such as aldehyde oxidase and the mitochondrial amidoxime reducing component 1 and 2, remain speculative [17,18]. There is literature on the potential benefits of inhibiting the molybdoenzymes aldehyde oxidase and xanthine oxidase for chronic disorders [19,20] and reactions catalysed by molybdoenzymes generate damaging reactive oxygen species such as hydrogen peroxide and the superoxide radical, even as they metabolise toxins [16,20]. In contrast, molybdenum-based nanoparticles and salts have been shown to act as antioxidant and cytoprotective agents [21,22,23,24], which may be independent of molybdoenzyme action. To date, however, the existing data has not been collated in terms of placental or gestational health. Therefore, this review examines the role of molybdenum in human health during pregnancy.

## 2. Molybdenum: Ubiquitous Yet Unknown

### 2.1. Discovery of Molybdenum and Chemical Versatility

**Figure 1:**
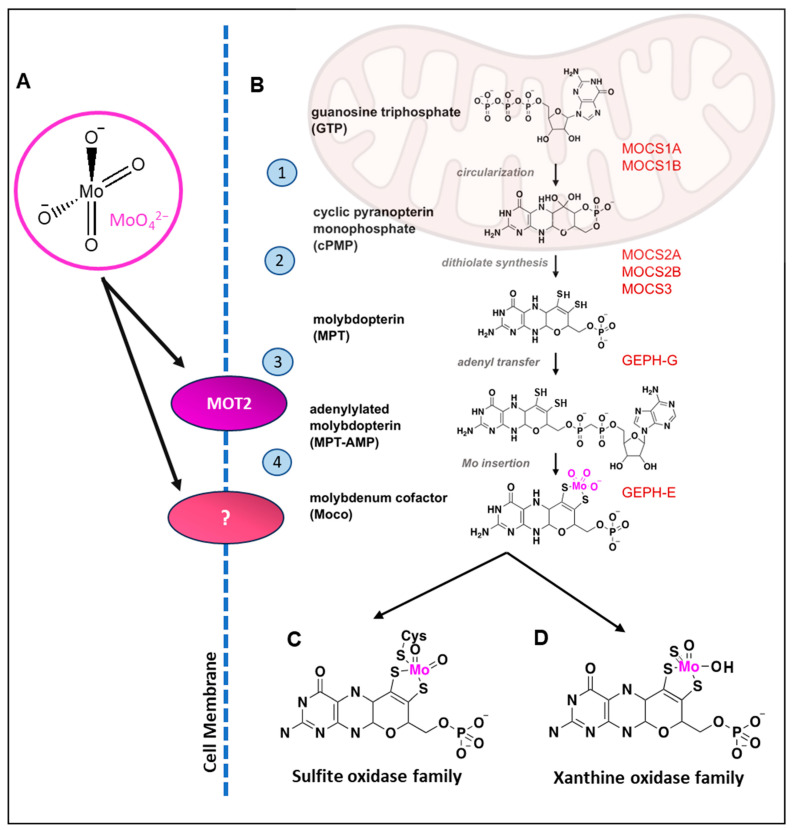
Molybdenum cofactor biosynthesis in human physiology. (**A**) Represents the structure of the MoO_4_^2−^ molybdate (VI) anion with tetrahedral formation. (**B**) Represents a simplified schematic of molybdenum cofactor biosynthesis. Moco is synthesized by a conserved biosynthesis pathway that is typically divided into four steps [28,30,31], whose main features are in italics, producing the biosynthetic intermediates (in black), and catalysed by the six associated proteins (in red) in humans [32,33,34]. Step 1 requires the conversion of 5′-guanosine triphosphate (GTP) to cyclic pyranopterin monophosphate (cPMP), a common step in all pteridine syntheses. MOCS1A and MOCS1B catalyse this process, with MOCS1A, as a member of the superfamily of S-adenosylmethionine (SAM)-dependent radical enzymes, catalysing the formation of protein radicals via [4Fe-4S] iron-sulfur clusters. GTP is thus converted by MOCS1A to 3′,8-cylodihydro-GTP, 3′,8-cH2-GTP (not shown), and then to cPMP by MOCS1B [29]. Both MOCS1A and MOCS1B transcripts contain a mitochondrial targeting sequence for translocation to the mitochondria, indicating cPMP is synthesised in the mitochondria and must be exported into the cytosol for subsequent steps [35]. In step 2, two sulfur atoms are transferred to cPMP to generate molybdopterin (MPT) dithiolate, by the MPT synthase enzyme complex which consists of two large (MOCS2B) and two small (MOCS2A) subunits [36]. MOCS2A requires adenylation and resulfuration by MOC3 for activation and transfer of the sulfur atoms [37,38]. MOCS2A then interacts with MOCS2B, which binds cPMP and produces MPT after the transfer of two sulfur atoms. In contrast to the other pteridine pathways (producing three-carbon side chains) MPT has a four-carbon side chain. Step 3 of Moco synthesis involves the adenylylation of MPT by the multifunctional protein gephyrin via the *N*-terminal G-domain (MPT adenylyltransferase, GEPH-G), followed by insertion of molybdenum by *C*-terminal E-domain (Mo insertase, GEPH-E) as the fourth step [33,39,40]. Transport of the molybdate anion across the cell membrane is necessary for completion of step 4 and is facilitated by molybdate transporters, including the Molybdenum Transporter 2 family [27]. Additional transporters are speculated, and depicted with a question mark. Steps three and four are sometimes combined in the literature as the third step of Moco synthesis [29]. Both adenylation of MPT and molybdenum ligation to MPT are magnesium dependent [39,41]. Subsequently, cysteine can be ligated to the molybdenum atom, losing one of its oxygen atoms as water in the process, generating (**C**) the molybdenum cofactor in enzymes of the sulfite oxidase family, with a protein-derived cysteine sulfur ligand [42]. Alternatively, a sulfur atom is bonded to the molybdenum atom, catalysed by the enzyme Moco sulfurase (HMCS), and generates (**D**) the functional molybdenum cofactor of the xanthine oxidase family, with an inorganic terminal sulfur ligand [42,43]. For further details of this process, please see [28,29,30,31].

Initially mistaken for lead upon its discovery in 1778, molybdenum (Greek “molybdos”, lead-like) has become recognised as an essential micronutrient for all multi-cellular eukaryotes—although the literature remains limited, and molybdenum relatively obscure as an area of study in human health [25]. Notably, molybdenum’s strength and corrosion resistance are vital in steel production, flame retardant products, and catalysts [26]. These unique attributes, determined by molybdenum’s redox chemistry, which facilitate its industrial importance, make it integral to all life and homeostatic functions. Molybdenum does not exist naturally as a free metal, with the bioavailable form of molybdenum occurring in soil and natural waters as the molybdate anion, MoO_4_^2−^ from molybdenum (VI) oxide (Figure 1A). The molybdate anion is transported into the cell via carrier proteins such as the Molybdenum Transporter 2 (MoT2) [27], and encompassed within the molybdenum cofactor (Moco), which is in turn integrated into molybdoenzymes for their functionality [28]. This is a complex process, and thoroughly explained elsewhere but briefly, Moco synthesis involves the interaction of six proteins, ATP, iron, and copper, in a conserved four-step process which culminates in the insertion of the molybdenum atom within a unique tricyclic pterin scaffold (Figure 1B) [28,29]. Moco is swiftly transferred to the requisite enzyme and buried deeply within the structure, and is hypothesised to be inserted prior to the completion of protein folding [28]. 

Most molybdenum-dependent reactions involve the transfer of a single oxygen atom to or from key metabolites, at a carbon, nitrogen or sulfur atom [44]. Two molybdoenzymes families which use molybdenum at their active site occur in humans, with one grouping including the Xanthine Oxidoreductase (XOR, also XO or XDH) and Aldehyde Oxidase (AOX) enzymes, and the second Sulfite Oxidase (SO) and (tentatively) the Mitochondrial Amidoxime-reducing Component 1 and 2 enzymes (mARC1/2). The main differences between these enzyme families arise in the choice of the fifth ligand, the fifth molecule attached to the central molybdenum atom, in coordination with the pterin-based scaffold of the molybdenum cofactor. The XO family use an inorganic “terminal sulfur” ligand (Figure 1D), which is essential for catalytic activity, while the SO (Figure 1C) family contain a protein-derived cysteine sulfur as the fifth ligand [45]. The mARC proteins remain difficult to place, as they contain a protein-derived cysteine as the fifth ligand, placing them as members of the SO family, while the coordination of the pyranopterin more closely mimics the XO family [46].

**Figure 2:**
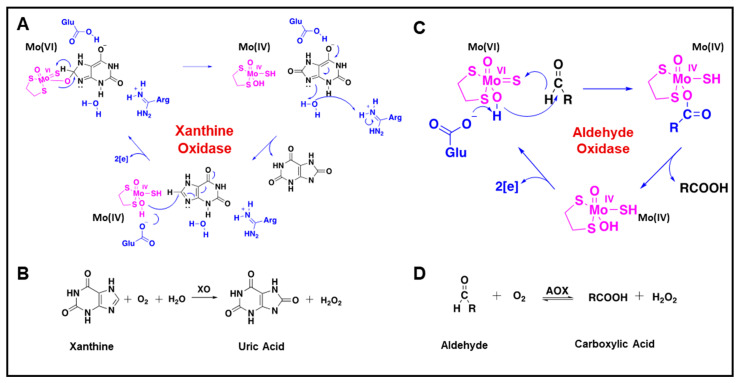
Overview of the molecular mechanism of the xanthine oxidase molybdoenzyme family. (**A**) Represents a simplified schematic of xanthine oxidase catalysing the reaction of xanthine to uric acid, through the action of Moco (in pink). Firstly, the proton from the Moco, Mo(VI), hydroxyl group passes to the Glu1262 amino acid residue of XO, with the resultant activated hydroxyl (–OH) group generating a nucleophilic attack on xanthine (in black). Then, a hydride is transferred from the tetrahedral intermediate to the sulfur atom of the Moco, reducing Mo(VI) to Mo(IV). Thirdly, uric acid is formed through its protonation by Arg881. Once this reaction is complete, FAD is reduced to FADH_2_ (not shown), oxidizing the Mo(IV) to its initial oxidation state of Mo(VI) [56,57]. Two reactive oxygen species (ROS) are generated in this reaction, superoxide anion (O_2_^•−^, also presented as 2[e], and hydrogen peroxide (H_2_O_2_—see (**B**)). (**C**) Simplified schematic of aldehyde oxidase catalysing the reaction of aldehyde (RCHO, in black) to the corresponding carboxylic acid (RCOOH, in black), through the action of Moco (in pink). In the first step, the site of oxidation is the electrophilic carbon of the aldehyde, which is attacked by the nucleophilic –OH ligand of the Moco (MoVI), aided by the Glu1270 residue (in blue). Hydride transfer to Moco’s sulfido ligand also occurs to form a transition state stabilised by hydrogen bonding from Glu1270 and Lys893 (not shown). In the catalytic cycle, molybdenum converts between the (VI) and (IV) valences, while FAD converts between the oxidised and reduced state. The 2FeS centre of the enzyme may mediate the transfer of electrons between the Moco and FAD, and serve as an electron sink [17,20]. Two reactive oxygen species (ROS) are generated in this reaction, O_2_^•−^, and H_2_O_2_—see (**D**).

Organisms use molybdenum’s chemical versatility to achieve specific enzymatic functions. Molybdenum, as part of the enzymatic active site, is reduced from (VI) to (IV) and oxidised from (IV) back to (VI) in multi-step processes that break down toxic substrates, generating reactive oxygen species [47,48] (Figure 2). Molybdoenzymes are widely expressed across numerous tissues due to their integral role in amino acid metabolism and catabolism of toxic substrates and pharmaceuticals, although the true complexity of their roles is yet to be elucidated [49]. To date, sulfite oxidase is the only molybdoenzyme that has been described as essential for human life. In humans, mutations to the sulfite oxidase gene result in retardation, seizures, and neonatal death [50]. Sulfite oxidase participates in the degradation of sulfurized amino acids such as cysteine and methionine, oxidising toxic sulfite to sulfate. Although deletion is not (immediately) lethal, xanthine oxidoreductase (also known by its isoform, xanthine oxidase, XO) is important for homeostatic control, with decreased xanthine oxidase leading to obstruction of the renal tract due to xanthine stone formation as a result of excess xanthine [51]. Paradoxically, deficiency of XO, hereditary xanthinuria, may be asymptomatic in some individuals [52]. If undetected, xanthine stones may lead to irreversible kidney damage. In contrast, increased xanthine oxidase activity produces excess uric acid which forms monosodium urate crystals within the joints, resulting in gouty arthritis and inflammation [53]. The catalytic versatility of XOR allows it to act both as an oxidase for purine-containing prodrugs, as well as a reductase for quinone drugs, and it can either activate or degrade drugs in vivo [54]. In addition, aldehyde oxidase and the recently discovered mARC enzymes are known for their roles in detoxification of xenobiotics and drug metabolism; however, their precise physiological functions and endogenous substrates remain speculative [18,20,55]. 

### 2.2. Bioavailability and Intake of Molybdenum

Typically, molybdenum is found in low quantities within soil (<10 mg/kg) and freshwater streams (median 0.5 μg/L) [58]. At an average concentration of 10 μg/L in salt water, molybdenum is the most abundant transition metal in open seawater [58]. In humans, molybdenum is primarily sourced from the diet, with staple foods such as legumes, grains, and liver being especially rich sources [59]. Concentrations per gram of fresh weight of soybeans and liver were calculated at 2.99 and 0.76 μg, respectively, in a Japanese study [60], while an American study found black-eyed peas and liver had molybdenum concentrations of 3.49 and 1.227 μg/g, respectively, more than sufficient to meet recommended molybdenum intake in a serving [61]. Concentrations of molybdenum within different food types range widely, and are dependent upon the alkalinity of the soil in which the food is grown and the water used for irrigation, with daily intake further modulated by dietary practices such as vegetarianism and seasonal food availability [59,62,63]. Once consumed, molybdenum is transported through the gastrointestinal tract via what was hypothesised to be a passive, non-mediated process, although the exact mechanism and location of the transporters remain unclear, and recent research indicates the existence of higher-affinity molybdenum transporters [27,64]. Absorbed molybdenum is rapidly detectable in blood, with a fraction reportedly binding to α2-macroglobulins, and to the cytoskeletal protein spectrin in the erythrocyte membrane, with the majority remaining unbound as molybdate [63,64,65,66]. The molybdenum cofactor, Moco, is sensitive to oxidation, so it is postulated to exist in a protein-bound state in the cell [28]. Although not detected in humans, a protein that binds to and protects Moco from oxidation, MCP1, has been identified in other organisms [67]. Absorbed molybdenum is distributed to various tissues, with Giussani concluding its primary storage to be the liver and kidney compartments, with concentration in the liver estimated to be 0.9–2.7 mg in adult males and 0.7–2.1 mg in adult females, based on the review of the existing data gathered from human autopsy studies [68]. Nakanishi and colleagues, in their analysis of molybdenum dynamics in a live cell model, furthermore surmised from existing data that animal tissues/cells hold a higher concentration of molybdenum (50–1000 ppb, 0.5–10 µM) than bodily fluids such as serum, urine, and milk (1–50 ppb, 10–500 nM) [60,69,70]. Analytical methods used in molybdenum content studies typically include inductively coupled plasma mass spectrometry (ICP-MS), inductively coupled plasma atomic emission spectrometry (ICP-AES), or instrumental neutron activation analysis (INAA), in which no distinction is made between total molybdenum and the molybdate anion [60,70,71,72].

The literature concerning molybdenum nutrition presents varied numbers. Mean daily intake of molybdenum was estimated as 82 μg/day for Australian women under thirty and 117 μg/day for Australian men in the same age group according to the 22nd Australian Total Diet Study, which specifically focused on dietary exposure to trace elements [73]. The European Food Safety Authority also collated studies throughout Europe over a period of several decades to examine dietary molybdenum exposure, with mean molybdenum intakes of adults ranging from 58 µg/day for women in Eastern Germany (1988 study) [74], to 79.6 µg/day for adults in Northern Italy (2008 study) [75] to daily intake per capita recorded at 157 µg/day in Sweden (2010 study) [63,76]. In rural communities in India, daily intake for men was calculated via a 24 h diet recall interview, at 230 µg/day [77], and 214 µg/person/day in a total diet study of the Japanese general population [78]. Differences in analytical methods may also account for this range, which include the market basket approach (analysing food representative of the average diet for any given demographic, and prepared for table consumption), the duplicate diet method (retaining an exact duplicate of all food and drink consumed during the study for analysis) and the total diet method (the collection of national and regional food types, with primary foods combined into composite samples and analysed). Most methods included procuring and preparing food as would occur in the home, with homogenisation and analysis performed using analytical methods such as ICP-MS, inductively coupled plasma optical emission spectroscopy (ICP-OES), or inductively coupled high-resolution mass spectrometry (ICP-HRS) [63,73,74,76]. In studies which included multiple demographics, women were noted to consume less molybdenum, consistent with lower food consumption overall in comparison to men [63,73].

### 2.3. Current Nutritional Parameters

Despite the wide range of intakes, all values were comfortably within accepted nutritional parameters for each country, as recommended molybdenum baseline requirements are variable among government health bodies. Seminal balance studies performed to assess the minimal requirement of molybdenum for health were performed by Turnlund and colleagues in 1995, who derived a value of 22 μg/day in a study of four male subjects; accounting for food bioavailability assessments and miscellaneous element losses, the estimated average requirement (EAR) in the US was raised to 34 μg/day [64,79,80]. Considering the low subject number and limitations of the study, the recommended dietary allowance (RDA) was further increased to 45 μg/day to accommodate the needs of 97–98% of individuals in the group, and with no data available at the time for women specifically, this value was applied to all adults [64]. This data set was used by the National Health and Medical Research Council of Australia, and the Ministry of Health, Labour and Welfare of Japan, amongst others, to set their own estimated average requirements and recommended intakes [81,82]. The UK Committee on Medical Aspects of Food Policy (COMA), as well as other central and northern European health organisations, did not set specific recommendations, but COMA recommended a safe intake range of 50–400 µg/day [63,83]. In comparison, the German, Austrian and Swiss (D-A-CH) Nutrition Societies set an adequate intake range of 50–100 µg/day [63,84]. 

Based on the existing studies and varied recommendations, it can be concluded that there is little debate regarding molybdenum deficiency or overexposure in human populations. Molybdenum is absorbed efficiently from aqueous preparations, with minor changes in absorption efficiency between doses ranging from 22 µg/day to 1500 µg/day, and no adverse effects reported in studies involving healthy adult men [80,85]. Uncommonly, absorption efficiency does not decrease with higher molybdenum intakes [79]. The literature varies on molybdenum absorption from food. Absorption of isotopically labelled molybdenum in composite meals was reduced in comparison to aqueous solutions (35–50% and 100%, respectively), in a study by Werner and colleagues, and a ten-fold reduction in molybdenum absorption was noted if ingested with black tea [86]. The food matrix was found to affect molybdenum absorption, with 86.1% of intrinsically labelled molybdenum absorbed from kale casseroles in comparison to 56.7% from soy casseroles, in one study of 12 young women with each meal containing ~100 µg [87]. Higher absorption rates at >90% have been recorded in a mixed Japanese diet study [71], and generally absorption of molybdenum is considered sufficiently high to meet and exceed recommended daily intake requirements. Plasma molybdenum reflects dietary intake, as does urinary molybdenum, which is the main excretion pathway from the human body [71,79,80,88]. Molybdenum content in faeces was used to calculate absorption. Urine molybdenum content is responsive to increased molybdenum intake [85,88], with 60% of molybdenum excreted via this pathway at very low dietary intake, and over 90% at the highest dose of molybdenum tested [79]. Both urine and serum therefore, may have utility as indicators of molybdenum status [63].

### 2.4. Rare, But Present: Molybdenum Deficiency and Overexposure

There is only one case recorded within the literature of dietary molybdenum deficiency, in a Crohn’s patient on total parenteral nutrition: they developed headaches, tachycardia, and night blindness, and biochemical analyses revealed low urinary sulfate and elevated plasma methionine concentrations, indicative of low molybdoenzyme activity [89]. The patient’s condition improved following treatment with ammonium molybdate. Molybdenum cofactor deficiency, meanwhile, is an autosomal recessive disorder, in which the biosynthetic pathway of de novo cofactor synthesis required for molybdoenzyme function is disrupted, due to mutations in one or more of the molybdopterin synthesising enzymes (Figure 1). Moco deficiency leads to excessive sulfite accumulation, neurological dysfunction and high 1-year mortality rates [90]. Roughly 100 cases of this condition have been found in the literature [91]. In cases of Moco deficiency due to MOCS1 mutations, the condition can be improved by substitution therapy with synthetic cyclic pyranopterin monophosphate, which was approved by the Food and Drug Administration in 2021 [92]. 

Overexposure of molybdenum in humans is equally rare; Kovalskii and colleagues described a case in Armenia where soil molybdenum was unusually high, resulting in estimated intake levels of 10–15 mg/day [25,93]. High levels of the molybdoenzyme xanthine oxidase and serum uric acid levels were reported, with symptoms of gout, aching joints, and hyperuricosuria observed [25,93]. Occupational molybdenum exposure is the main avenue of toxicity reported in the literature—one case study made a tentative link between a male patient’s aching joints, hyperuricemia, and gout symptoms and his occupation in the production and maintenance of molybdenum-lined vacuum furnaces [94]. During an exposure-free period, his symptoms abated, and they relapsed once he returned to the high-molybdenum environment. Workers within a molybdenum roasting plant exposed to high concentrations of molybdenum dust received a daily body burden of 10.2 mg of molybdenum per 8 h shift, and reported joint pain, backaches, headaches, and heightened molybdenum and uric acid in serum and urine, although the high worker turnover rate limited the investigation and conclusions [95]. There were no fatal cases associated with exposure, but the data was considered inadequate for establishing adverse effect levels or a tolerable upper intake level. 

In pregnancy, there are few studies of high dosage supplementation. Administration of a supraphysiological dose of molybdenum (15 mg/day with ferrous sulfate) to a pregnant human cohort has been conducted with no toxic effect reported [96]. Molybdenum toxicity has been evaluated in rats and mice. High molybdenum diets at dosages of 10–100 mg/L sodium molybdate in rats resulted in growth retardation, spinal cord abnormalities, and prolonged oestrus [97]. Based on this study by Fungwe and colleagues, the no-observed-adverse-effect level (NOAEL) was calculated as 0.9 mg/kg/day of molybdenum, and a tolerable upper intake level of ~2000 μg/day was set in humans, equivalent to 0.03 mg/kg BW per day, assuming 68.5 kg BW [64,97]. In contrast, a study providing a daily dosage of 8 mg/day (8000 μg/day) molybdenum for 12 days reported no adverse effects in the healthy, human study population (*n* = 24) [98]. A case of acute exposure, in which a single dose of sodium molybdate corresponding to 62.5 mg/kg was ingested also showed no clinical manifestations [99], indicating that there is a need for further studies to establish a tolerable upper intake level and a no-observed-adverse-effect level (NOAEL) with humans at acute and chronic exposure.

### 2.5. Gaps in the Research—Molybdenum in Pregnancy

The capacity of the human body to adapt to great variations in molybdenum availability, and the rarity of reports on deficiency, may be responsible for the limited research on molybdenum nutrition in pregnancy. No direct data were used to generate the recommended daily allowance of molybdenum during pregnancy—the value of 50 µg/day was determined by extrapolating from the existing RDA set for non-pregnant women, with the addition of 16 kg to the reference weight, based on an American study of pregnancy outcomes [64,81]. In recent years, there has been increased scrutiny of micronutrient intakes during pregnancy, given the (a) high-fat, low-quality diet often said to be calorie dense and nutrient poor, which has become common in high-income countries and termed the Western diet, (b) the resultant increase in obesity and micronutrient deficiencies during pregnancy, and (c) the lack of adequate dietary changes following conception, to meet foetal demand [11,100]. However, despite these trends, there has been no modification to the original values presented as adequate for molybdenum intake, and reviews of maternal nutrition during gestation frequently do not mention molybdenum at all [11,101]. 

Molybdenum transport and uptake in higher-order organisms remains understudied in comparison to bacteria [31]. Tejada-Jimenez and colleagues discovered a common transporter shared between the algae *Chlamydomonas reinhardtii* and higher-order animals including humans, termed the Molybdenum Transporter 2 (MoT2, or MFSD5), with conserved motifs that separate this protein family from the more established prokaryotic Molybdenum Transporter 1 (MoT1) system [27]. In algae, activation of this gene was observed in environments with low molybdenum concentrations, while in humans, MoT2 transcript has been detected in multiple tissues, with higher accumulation noted in the cervix, amongst other locations [27]. Although this high-affinity uptake system is hypothesised to be the main molybdate importer for the cell, alongside a potential non-specific avenue via sulfate transporters, its location within the cell remains undetermined [31], and it has not been linked to placental transport or gestation, in the literature. Nakanishi and colleagues were the first to examine molybdate dynamics in animal cells (HEK-293T cell line) through the creation of a real-time fluorescence detection system, a Förster resonance energy transfer (FRET)-based nanosensor composed of cyan fluorescent protein, yellow fluorescent protein, and the bacterial molybdate-sensor protein ModE [69]. Overexpression of MoT2 in this system accelerated molybdate intake into the cell-but knockdown of this gene did not affect intake, with a novel, and currently unidentified, oxalate-sensitive and sulfate-resistant transporter(s) evidenced to also be responsible for molybdate transport [69]. 

There is scarce literature on the modulation of molybdenum transport in pregnancy; one study examining micronutrient levels between the umbilical artery, vein, and maternal vein of healthy pregnant women concluded that molybdenum appeared to passively diffuse across the placenta [102]. This has since been contested in a study which found elevated molybdenum concentrations in umbilical cord tissue in comparison to foetal membranes and the placenta [14]. The sodium-dependent sulfate transporter, Nas2, can transfer oxyanions of selenium, chromium, and molybdenum, with expression primarily found in rat and human trophoblast (placental) cells [103]. The enzymatic activity of the molybdoproteins, xanthine and sulfite oxidase is influenced by molybdenum intake, or following an increase of tungsten in the diet, which antagonises molybdenum by competing for insertion within Moco, in animal studies [89,104,105]. While it may be argued that these are extreme examples of deficiency, a lack of clinical symptoms does not mean underlying biochemical changes are not occurring within the tissue, particularly in situations of heightened requirements and oxidative stress, such as pregnancy. Tentative links between low molybdate bioavailability in the soil, and an increased incidence of chronic diseases such as gastric cancer and Keshan disease (a form of cardiomyopathy) have previously been reported [106,107], with the pathophysiology of both modulated by oxidative stress and antioxidant dysfunction. Therefore, while deficiency to the point of severe clinical symptoms or fatality may not be occurring, *insufficiency* may be influencing long-term gestational complications. Insufficiency is not uncommon in the literature in relation to a variety of micronutrients, and therefore increases ambiguity [108,109,110]. There is an opportunity and impetus to examine molybdenum critically in a similar fashion to selenium, a micronutrient required in trace quantities, and whose insufficiency is also implicated in disorders such as cancer, and gestational complications [111,112]. Selenium is a key player in multiple antioxidant pathways, and in the selenoprotein family of over 20 enzymes. Low selenium levels and reduced selenoprotein enzyme activity have been found in pre-eclamptic pregnancies, and are associated with other complications of gestation including growth restriction and preterm birth [111]. Selenium supplementation was found to increase the activity of antioxidant enzymes with selenium cofactors, and confer a protective effect in trophoblast cell lines following mitochondrial-induced oxidative stress [113]. Furthermore, considering its narrow therapeutic range and the current research around the justification of its supplementation in pregnancy [114], selenium provides an important precedent for other micronutrients such as molybdenum to be investigated in the context of pregnancy, placental health and oxidative stress.

## 3. Molybdenum Exposure during Pregnancy and Disease

### 3.1. The (Understandable) Overexposure Bias

It is apparent in the literature on molybdenum supplementation that there is a focus primarily on extreme, non-physiological exposure levels. The dosages reported in the literature were typically in the tens to hundreds of milligrams per kilogram of body weight range, magnitudes above the usual consumption concentrations and recommended daily intake, with the aim of understanding the overexposure risk [97,115]. The underlying mechanisms of the adverse responses to high exposure to molybdenum were hypothesised, but not explored in depth. They included molybdenum-induced copper depletion, which could potentially affect downstream processes such as antioxidant enzyme function and mitochondrial respiration, altered purine metabolism as a result of increased molybdoenzyme activity, and an overall increased oxidative stress as a result. Considering the focus of this review on pregnancy and dietary intake, and to reduce the complexity of the data studied, the subsequent focus will be on supplementation with molybdenum salts, or biofortification in food, which more closely mimic physiological intake. 

### 3.2. Molybdenum as a Mediator of Hyperglycaemia and Hyperlipidemia, and Its Role in GDM

Molybdenum salts have significant cytoprotective, antioxidant, and insulin-mimicking effects in vitro and in vivo in a range of animal models. Sodium molybdate orally administered at 100 mg/kg significantly reduced total cholesterol, triglyceride and low-density lipoprotein levels, and exhibited a synergistic beneficial effect when combined with the lipid-lowering drug atorvastatin in a diet-induced hyperlipidemic hamster model [116]. Sodium molybdate supplementation in drinking water, estimated to equate to 50 mg Mo/kg/day for the highest dose, likewise reduced hepatic triglyceride levels in a non-alcoholic fatty liver disease mouse model [117] and, in a complex with ascorbic acid, reduced levels of blood glucose and lipids [118]. Ozcelikay and colleagues found sodium molybdate supplementation (0.4–0.5 g/L in water and 0.75–1.25 g/kg in food) significantly reduced plasma glucose levels in diabetic rats with no effect on circulating insulin levels, restored the expression and activity of two major glycolytic enzymes in the liver (glucokinase, GK and L-type pyruvate kinase, L-PK) and reduced the elevated expression and activity of a major gluconeogenic enzyme, PEPCK, to near control levels in the diabetic group [119]. The expression and activity of key enzymes of lipogenesis, fatty acid synthase (FAS), and acetyl-CoA carboxylase (ACC), were modulated by molybdenum supplementation, in liver but not adipose tissue [119]. Molybdenum was postulated to exert effects at the pre and post translational level, due to differences in expression of mRNA versus enzymatic activity, and its mechanism of action was concluded to occur at post binding and probably post receptor kinase steps, as the insulin receptor number and tyrosine kinase activity were unaffected [119]. Overall, oral sodium molybdate supplementation reduced gluconeogenesis through reduction of the enzyme PEPCK. Dysregulated gluconeogenesis is a noted contributor to the diabetic phenotype, and the effects of molybdenum supplementation on this process, and the diabetic phenotype overall, have been reproduced in the literature [120,121]. While the concentrations of these dosages are still magnitudes higher than the recommended daily intake for humans, no adverse effects were noted in the experimental animals. 

The insulin and antidiabetic effects of molybdenum have been observed in rodent models with the related trace metals tungsten and vanadium, with similar reductions in blood glucose levels, PEPCK activity, and increased activity of glycolytic enzymes GK and PK [122,123]. Vanadate, tungstate and molybdate are anions with similar chemical properties and geometry to those of the phosphate ion. Phosphate and its metal analogues, including molybdate, can inhibit the activity of protein tyrosine phosphatase 1B, which is a prominent negative regulator of insulin and leptin signalling pathways, and may be a molybdoenzyme-independent mechanism of action for the anti-diabetic properties of molybdenum [124,125]. In humans, vanadium was also found to increase intracellular free magnesium levels in erythrocytes in vitro in a similar manner as physiological doses of insulin [126], which may provide an additional pathway through which the chemically similar molybdate anion could change the intracellular environment. 

With molybdenum established in the literature as an insulin mimetic and therapeutic agent in metabolic disorders, its function in human populations has also been assessed (Table 1). In a case-control study in a Chinese adult population, an inverse relationship existed between plasma molybdenum and the risk of metabolic abnormalities including hyperglycaemia, hypertension and abdominal obesity, independent of established risk factors for metabolic syndromes, and concentrations of other essential nutrients [127]. Median plasma concentrations of molybdenum were 1.24 μg/L and 1.46 μg/L for the ‘metabolic syndrome’ group and the control group, respectively, with increased plasma molybdenum levels associated with decreased risk of not only a broadly metabolic syndrome, but specifically hypertriglyceridemia, hyperglycaemia, hypertension, low high-density lipoprotein levels, and abdominal obesity. This relationship broadly levelled off around plasma molybdenum concentrations of 2.0 μg/L [127]. This dose-dependent effect and plateau may be reflective of the narrow beneficial range observed in previously reported animal studies, which may also explain some of the conflicting results around molybdenum plasma concentrations. Ajibola and colleagues, for example, found significantly increased plasma molybdenum in diabetic patients in comparison to controls in a small Nigerian population study, hypothesising that the increased levels of Mo may be due to increased uptake in response to carbohydrate metabolism deregulation and the oxidatively stressful phenotype of diabetes [128]. Flores and colleagues, meanwhile, hypothesised that the observed increase in plasma molybdenum in an older, small diabetic cohort was due to inadequate (beneficial) elimination of the element from circulation, corresponding to decreased urinary molybdenum content in the diabetic patients in comparison to the healthy group [129]. Two US-based population studies found a link between heightened urinary molybdenum concentrations and increased fasting plasma glucose [130] and insulin resistance and diabetes [131], which was contradicted by a study in a multi-ethnic, perimenopausal cohort, who found urinary Mo concentration was significantly inversely associated with insulin resistance [132]. A second study by this group using a similar cohort did not find evidence for an association between urinary Mo and diabetes risk [133]. The diversity of results could have multiple causes, including sex and ethnic differences in molybdenum metabolism, which may be exacerbated in underpowered, small population studies, and by the severity of the metabolic disease (Table 1). Furthermore, there are acknowledged limitations to urinary ‘spot’ sampling in the analysis of global molybdenum status, which can fluctuate rapidly based on recent intake, and may not be representative of prolonged molybdenum exposure. It is currently unclear whether urinary molybdenum content is indicative of reduced molybdenum uptake to bodily tissues, or increased molybdenum exposure. More recently, the literature has begun to explore intracellular molybdenum content alongside blood and urine; Toro-Roman and colleagues found the intracellular content of platelets and erythrocytes substantially higher than concentrations in plasma, but it is not known whether molybdate, the molybdenum cofactor, or another molybdenum complex was detected [134]. Lastly, a two-arm study investigating the effects of a 12-day nutritional intervention with molybdenum biofortified lettuce (8 mg/100 g fresh weight consumed per day) significantly reduced fasting glucose, insulin and insulin resistance, with no change in urinary output of molybdenum between groups, which may be due to the rapid clearance of molybdenum shortly after ingestion. This study, although small (*n* = 24), provided potential proof of concept for molybdenum fortification in a human population [98].

These contradictory data sets are reflected in the literature of molybdenum in pregnancy, across two methods of analysis, ICP-MS and AAS (Table 2). Molybdenum status in the late first trimester of pregnancy was significantly inversely correlated with high glucose levels in the late second trimester, in a prospective study of a healthy pregnant cohort (*n* = 1857) [135]. Median plasma molybdenum values in this cohort were 1.9 μg/L, with a 50% increase in molybdenum associated with a 14% reduced risk of abnormal glucose values from the gestational load test [135]. This was confirmed by a second paper from this research group, which compared three different statistical models to calculate associations between molybdenum plasma levels in early pregnancy and glucose levels in the second trimester; all models were consistent in finding a negative association between Mo levels and glucose levels, although the association for Mo was weaker in Bayesian kernel machine regression (BKMR) than in the generalised adaptive model (GAM) or the adaptive least absolute shrinkage and selection operator (LASSO) [136]. A study investigating supplementation use in a small Australian cohort however, found no evidence of a relationship between serum or dietary levels of Mo and GDM [137]. These data sets are the most recent, and are preceded by a collection of papers by Al-Saleh and colleagues, which are uncommon in their study of umbilical molybdenum status, and may better reflect foetal exposure. However, subject numbers were low (*n* < 32) across all studies. Al-Saleh and colleagues found no statistically significant difference in maternal molybdenum serum concentrations between healthy and gestational diabetes pregnancies at term delivery, while umbilical vein and artery concentrations of molybdenum were significantly higher in the GDM group than in the control [138]. Al-Saleh and colleagues, furthermore, found that in the obese GDM pregnancies significantly higher concentrations of maternal vein and umbilical artery molybdenum concentrations were found, in comparison to obese controls [139]. Umbilical vein and artery concentrations of molybdenum were also higher in insulin-dependent diabetic women who became pregnant, in comparison to healthy controls, although maternal molybdenum content and maternal/foetal ratios of the element in maternal and umbilical veins were not considered significantly different [140]. Concentrations of molybdenum across the different pathologies and papers within this set typically ranged from 11 to 18 μg/L, significantly higher values than those reported by Zheng and colleagues for the first trimester of pregnancy. Taken together, these data suggest that molybdenum transport into foetal compartments might be altered in pathological states in comparison to the more passive transport observed in healthy controls. 

The heightened inflammation, oxidative stress and reduction in antioxidant enzymes that accompanies gestational diabetes mellitus [141] might be ameliorated by a molybdenum supplementation, if we look to animal model data—but current human studies are few, and lack continuous data across the gestational period (Table 2).

### 3.3. Molybdenum and Antioxidant Activity 

Although the mechanism remains unknown, when molybdate salts—the bioactive form of molybdenum—are administered, studies have observed a modulation of other antioxidant enzymes. Potassium and ammonium molybdate salt supplementation (15 mg Mo/BW per day) raised the activity of the antioxidant enzymes superoxide dismutase (SOD), catalase (CAT), and glutathione peroxidase (GPx) in brown goats exposed to cadmium and copper pollution, and modulated the animals’ immunological response to heavy metal poisoning via serum pro-inflammatory cytokine activity and immunoglobulin content [142]. Sodium molybdate, a low-toxicity and highly soluble compound, has been shown to significantly increase the activity of the antioxidant enzymes superoxide dismutase (SOD) and catalase (CAT) in rat hepatic cells at concentration ranges of 50–200 mg/kg BW, and to confer significant reductions in cell necrosis, inflammation and fibrosis, following induced liver damage [23,24]. While treatment dosages were acknowledged to be above the equivalent intake for humans, no adverse health outcomes were noted, even at the highest concentrations. In a mouse model of non-alcoholic fatty liver disease (NAFLD), sodium molybdate was noted to decrease levels of two oxidative damage markers: malondialdehyde (MDA) a lipid peroxidation product, and oxidised protein in diseased animals, at two concentrations (0.3 g/L, and 1 g/L sodium molybdate), estimated to equate to 50 mg Mo/kg/day for the highest dose [117]. SOD activity was likewise significantly increased at both concentrations in comparison to untreated diseased animals [117]. Sodium molybdate supplementation at 100 mg/kg, both with and without the lipid-lowering drug atorvastatin, also increased activity of the plasma antioxidant enzymes GPx and CAT, and reduced plasma lipid peroxide levels in the diet-induced hyperlipidaemic hamster model [116]. 

Zhang and colleagues found molybdenum had a dose-dependent antioxidant effect in mice; sodium molybdate ingested at dosages of 5 mg/L (water ad libitum) by healthy adult female mice significantly improved oocyte morphology and, ovulation number, and increased activity of both SOD and GPx at 10 mg/L [143]. At concentrations of 20 mg/L and above, however, the activity of SOD and GPx was significantly decreased, and morphologically abnormal organelles, including mitochondria, were observed [143]. Dose-dependent changes were recapitulated in vitro in mouse pre-implantation embryos, where a mild, non-significant beneficial effect was observed in cultures of 5 mg/L sodium molybdate, while concentrations of 40 µg/mL and higher significantly decreased the number of morphologically normal embryos and increased the incidence of cytoplasmic fragmentation and developmental arrest [115]. Sperm quality and the testicular oxidative environment were likewise improved with the addition of sodium molybdate, with a supplementation of 25 mg/L in mouse drinking water significantly improving sperm motility and, concentration, and decreasing the percentage of morphologically abnormal sperm improvements which correlated with increased activity of SOD and GPx [144]. These results were inverted with increased oxidative stress at a higher (100 mg/L) molybdate supplementation [144], indicative of a narrow therapeutic range for molybdenum salts, at least in certain animal models. It is pertinent that most of the literature examining the effect of molybdate supplementation does not focus on molybdoenzyme activity or expression, and therefore no conclusions can be made on specific downstream effects of supplementation on molybdoenzymes directly. An exception to this is the study performed by Lee and colleagues, which found that the activities of the molybdoenzymes Xanthine Oxidase, Aldehyde Oxidase, and Sulfite Oxidase, were not influenced at 0.3 g/L, or 1 g/L sodium molybdate supplementation in the NAFLD mouse model, although AOX activity was significantly decreased, and sulfite oxidase and XDH activity was significantly increased in the NAFLD animals in comparison to the control [117]. Moreover, the mRNA expression of cytoplasmic Cu/ZnSOD (SOD1), mitochondrial MnSOD (SOD2), and extracellular Cu/ZnSOD (SOD3) were not altered with sodium molybdate supplementation, despite the heightened activity of the enzymes [117]. In this instance it could be concluded that the molybdenum-induced alleviation of lipid accumulation and the mobilisation of antioxidant enzymes is not modulated, or not directly through the action of molybdoenzymes. The molybdoenzymes were assayed using their known substrates, aldehydes and sulfites, which may not accurately represent their capacity to metabolise other substrates within that tissue, such as nitrite to nitric oxide [145]. Furthermore, molybdate, given its unique redox chemistry and circulation in the blood and tissues, may act as a ROS scavenger itself, with molybdenum-based nanoparticles used as ROS scavengers in the literature, though this is speculative [21,22]. Moreover, the anion vanadate, which shares similar chemical geometry with molybdate, has been found to modulate intracellular free metal content [126]. It is possible that molybdate operates in a similar fashion to vanadate in modulating intracellular magnesium, which is a known antioxidant and anti-inflammatory agent. The relationship between molybdenum and other essential trace metal nutrients such as copper should also be considered, and may influence antioxidant signalling pathways, alongside the action of molybdoenzymes. 

### 3.4. Molybdenum and Foetal Development

Studies of the association between in utero exposure to molybdenum and adverse gestational outcomes have produced varied and contradictory results (Table 3 and Table 4). 

A positive correlation was found by Al-Saleh and colleagues, in a healthy term cohort, between maternal serum molybdenum status and placental weight, but no correlation was noted with birth weight, and no comment was made on the foetal/placental weight ratio, to estimate placental efficiency [102]. The relationship between circulating molybdenum concentrations (but not urinary) and placental weight was supported in a second-trimester Australian cohort study by McKeating and colleagues [146]. A small (*n* = 56) first-trimester pilot study examining Mo in urine similarly found no correlation with second-trimester anthropometric measures, or term birth weight [147]. Maternal urinary levels of molybdenum were negatively associated with foetal abdominal circumference (significantly) and estimated foetal weight, as based on ultrasound parameters, in a second-trimester cohort, with a more pronounced association found in pregnant women with relatively lower copper levels, but which was non-significant [148]. A third-trimester study in a healthy pregnant population found the z-scores of foetal parameters such as femur length, and, less dramatically, abdominal and head circumference, increased with higher molybdenum concentrations in maternal urine [149]. Concerning gestational complications, broadly, molybdenum status, as evaluated from maternal urine, serum, and plasma from existing second- and third-trimester studies, was not associated with preterm-birth, small-for-gestational-age (SGA), or pre-eclampsia outcomes [137,146,150,151,152].

**Table 3:** Selected studies on Molybdenum associations with foetal growth parameters and gestational complications in pregnancy.

Authors	Method of Detection	Population Number	Population Details	Matrix	Country	Results
Al-Saleh et al., 2004 [102]	AAS	39 healthy non-obese subjects	Late third trimester (39.2 + 0.3 weeks)	Blood/serum	Kuwait	Significant positive correlation was found between maternal and umbilical cord levels of Mo, and placental weight. Maternal blood Mo did not correlate with birth weight.
Goodrich et al., 2019 [147]	ID LC-MS/MS	56 healthy subjects	First trimester	Spot urine	US	No association found between urinary Mo in 1st trimester and 2nd trimester foetal biometrics or birth weight at term.
Kim et al., 2018 [150]	ICP-MS	390 subjects (99 preterm birth, 291 control)	Early third trimester (median 26 weeks)	Spot urine	US	No significant association found between urinary Mo and risk of preterm birth.
Kim et al., 2020 [149]	ICP-MS	390 healthy subjects	Early third trimester (median 26 weeks)	Urine	US	Positive association between urinary Mo and foetal z scores, inc. femur length (significant), abdominal circumference and head circumference.
Kot et al., 2019 [14]	ICP-AAS	83 healthy subjects	Late third trimester (39 ± 1.8 weeks)	Umbilical, placental and foetal membrane tissue	Poland	Negative association found between placental Mo concentration and placental width.
McAlpine et al., 2019 [137]	ICP-MS	127 subjects (89 used supplements, 38 did not)	Late second and early third trimester, 180–210 days gestation (25–30 weeks)	Fasting serum	Australia	No significant association found between Mo in serum or dietary levels, and the incidence of negative outcomes (low birthweight infants, pre-term birth, hypertensive disorders).
McKeating et al., 2021 [146]	ICP-MS	<128 subjects, crossover (10 with SGA, 18 with low placental weight, 13 preterm birth, 87 controls)	Second trimester (18 weeks)	Plasma, urine	Australia	Plasma (but not urine) Mo concentrations were significantly lower in pregnancies with low placental weight in comparison to controls. Plasma Se:Mo ratio had 87.3% predictive capability for determining placental weight.
McKeating et al., 2021 [151]	ICP-MS	328 samples (38 who developed PE, 91 who delivered with SGA, 193 healthy controls)	Late third trimester (36 weeks)	Plasma	Australia	No significant differences found between plasma Mo of controls, and negative outcome (pre-eclampsia and small-for-gestational-age) groups at 36 weeks. An increase in plasma Mo was noted with PE outcome (non-significant).
Shirai et al., 2010 [152]	ICP-MS	78 healthy subjects	Variable collection (9–40 weeks)	Spot urine	Japan	No significant association found between urinary Mo and birth size.
Zhao et al., 2021 [148]	ICP-MS	220 subjects	Second trimester (24.9 ± 0.8 weeks)	Spot urine	China	Negative association found between urinary Mo levels and foetal AC (significant) and EFW during the second trimester of pregnancy.

Abbreviations: AAS, atomic absorption spectrophotometry; AC, abdominal circumference; EFW, estimated foetal weight; ID LC-MS/MS, isotope dilution liquid chromatography-tandem mass spectrometry; ICP-AAS, spectrophotometric atomic absorption in inductively coupled argon plasma; ICP-MS, inductively coupled plasma mass spectrometry; PE, pre-eclampsia; Se, selenium.

A recent review by Huang and colleagues [153] reported significantly increased concentrations of molybdenum in maternal serum [154], maternal hair [155], umbilical cord tissue [156] and amniotic fluid [157] from healthy controls in comparison to pregnancies affected by neural tube defects (NTDs) (Table 4). Increased concentrations of molybdenum in maternal serum were associated with reduced risk of neural tube defects in the same study, with no correlation between molybdenum content and gestational age [154]. However, no evidence of a relationship between molybdenum and NTD development was found in a second, smaller study from this group [158]. Likewise, Liu and colleagues found that univariate logistic regression analysis showed that higher levels of Mo in umbilical tissue were associated with a lower risk of NTDs, but this protective effect was not observed following adjustment for confounders, notably gestational age [156]. Conversely, Yin and colleagues found a negative correlation between placental molybdenum concentrations and gestational age, and significantly higher median molybdenum concentrations found in the placental tissue of NTD-affected pregnancies in comparison to controls [159]. In this cohort, molybdenum concentrations were associated with increased risk of NTDs using a multivariable logistic regression model, but this relationship was not observed in a BKMR model with other elements fixed at the 25% and 50% percentile [159]. 

**Table 4:** Selected studies on molybdenum associations with neural tube defects in pregnancy.

Authors	Method of Detection	Population Number	Population Details	Matrix	Country	Results
Liu et al., 2020 [156]	ICP-MS	332 subjects (166 with NTD-affected pregnancies, and 166 controls)	Collection across first, second and third trimesters	Umbilical cord tissue	China	Median concentrations of Mo in umbilical cord tissue were significantly higher in controls than NTD-affected pregnancies. Mo concentration was significantly correlated with gestation in NTD cases. No relationship between Mo and NTD risk observed when adjusting for confounders.
Ovayolu et al., 2019 [157]	ICP-MS	75 subjects (36 with NTD-affected pregnancies, and 39 controls)	Second trimester (NTD: 21.6 ± 6.6 weeks, controls: 19.6 ± 2.4 weeks)	Amniotic fluid	Turkey	Mean concentrations of Mo in amniotic fluid were significantly higher in controls than NTD-affected pregnancies.
Tian et al., 2021 [154]	ICP-MS	750 subjects (273 with NTD-affected pregnancies, and 477 controls)	Collection across first, second and third trimesters	Serum	China	Median concentrations of Mo in maternal serum were significantly higher in controls than NTD-affected pregnancies. Mo was found to have a significant protective effect against NTDs.
Tian et al., 2022 [158]	ICP-MS	213 subjects (99 with NTD-affected pregnancies, 114 controls)	Collection across first, second and third trimesters	Serum	China	No significant difference between median concentrations of Mo in maternal serum from healthy controls and NTD-affected pregnancies.
Yan et al., 2017 [155]	ICP-MS	452 subjects (191 with NTD-affected pregnancies, 261 controls)	First trimester (4 weeks preconception to 8 weeks post)	Hair	China	Mo concentrations in maternal hair samples from healthy controls were significantly higher than in NTD-affected pregnancies, and specifically spina bifida-affected pregnancies. When adjusting for confounders, Mo hair content was inversely associated with NTD risk. Mo deficiency was associated with increased risk of NTD subtypes anencephaly and spina bifida.
Yin et al., 2020 [159]	ICP-MS	1001 subjects (408 with NTD-affected pregnancies, and 593 controls)	Collection across first, second and third trimesters	Placental tissue	China	Median concentrations of Mo in placental tissue were significantly higher in NTD-affected pregnancies in comparison to controls. Mo concentrations were significantly higher in females than males. Increased Mo concentrations were associated with higher risk of NTDs in multivariable logistic regression model, but was close to null in BKMR model.

Abbreviations: BKMR, Bayesian kernel machine regression; ICP-MS, inductively coupled plasma mass spectrometry; NTD, neural tube defects.

As none of these papers include biochemical data, it is difficult to assess the underlying pathways for these associations. Two recent reports found urinary molybdenum concentrations to be positively associated with lipid peroxidation biomarkers 8-isoprostaglandin F2α (8-isoPGF2α) and the oxidative DNA damage biomarker 8-hydroxydeoxyguanosine (8-OHdG) in the third trimester [160,161]. 

The relatively small and homogenous populations, and lack of data over the full course of pregnancy for the majority of cohorts studied may not provide a true reflection of molybdenum status in the general population, particularly via urine sampling. Unlike urine, maternal hair could provide exposure data over an extended period of time, and is less invasive to obtain and more easily transported than maternal blood, amniotic fluid or tissue. However, amniotic fluid or umbilical and placental tissue may more accurately represent foetal molybdenum status. An increased demand for molybdenum with gestational time course has been indicated within and between some studies with regards to circulating maternal molybdenum concentrations, and concentrations in certain foetal tissues. Molybdoenzyme function should also be considered within this increased demand, and would be affected by molybdenum insufficiency. However, the data are inconsistent, and further confounded by variabilities in the sample matrix, collection, and methodologies. 

A potential protective window of effect may also occur in the first trimester of pregnancy, considering the first-trimester studies of metabolic syndrome risk [135,136] and the body of literature investigating neural tube defects [154,155,157] in relation to molybdenum. Neural tube defects are caused by a failure of the neural tube to fuse by the fourth week post fertilisation (~28 days) [162].

## 4. The Role of Molybdoenzymes in Physiology

In addition to drug metabolism, molybdoenzymes are involved in oxidant biology, through the generation of reactive oxygen species (ROS) (Table 5) which can affect cellular pathways and viability through multiple mechanisms. Although often seen as negative, ROS are integral in mediating signalling pathways in cell growth, differentiation, and tissue repair [141,163]. In excess however, ROS can cause cellular injury, oxidise lipids and damage proteins, when exceeding antioxidant capacity, a physiological status referred to as oxidative stress. Oxidative stress is known to play a role in the pathogenesis of multiple diseases, including atherosclerosis, heart disease and cancer [164]. With specific relevance to gestational complications, ROS have been implicated in the aetiology of IUGR, pre-eclampsia, and accelerated dysfunction of the placenta [165], which may be further modulated by the ROS-producing molybdoenzymes (Table 5). 

The potential pathology-inducing action of molybdoenzymes is complicated by their secondary role as nitrite reductases, i.e., proteins which reduce nitrite to nitric oxide (Table 5) [145]. Nitric oxide is a low-molecular-weight and reactive free radical, and beneficial at low physiological concentrations by mediating mitochondrial respiration, calcium flux, vasodilation, and inflammation [166]. At high concentrations, or in the presence of ROS, nitric oxide can be cytotoxic and pro-inflammatory, and high nitric oxide levels have likewise been implicated in the pathogenesis of metabolic and cardiovascular diseases [167]. Nitric oxide is also a critical vasodilator in placental tissue, and its production is necessary for implantation, placental perfusion, angiogenesis, and remodelling of the spiral arteries required for adequate maternal blood flow through the placenta. Nitric oxide dysfunction is established as a cause of intrauterine growth restriction and hypertensive disorders of pregnancy [168]. The complex interplay between molybdoenzymes as toxin-metabolising and ROS-generating proteins, alongside their secondary role as generators of nitric oxide, should be taken into consideration with regards to the design of future clinical trials with molybdenum supplementation, or molybdoenzyme inhibition therapeutics.

**Table 5:** Molybdoenzyme location, substrates, and related pathologies.

Molybdoenzyme	Tissue Locations	Cellular Compartments	Substrates (Not Exhaustive)	Products (Not Exhaustive)	Associated Reactive Species	Pathologies	Pregnancy-Related Research
Aldehyde Oxidase	liver, gut, lungs, brain, adipose tissue, skin, placenta [17,20]	Cytosol [17]	organic aldehydes, azaheterocycles, exogenous toxins (e.g., vanillin), nitrite, prodrugs [17,20]	carboxylic acid, lactams, vanillic acid, nitric oxide [17,20,145]	O_2_^•−^, H_2_O_2_ [169]	Implicated in development of obesity, cancer, lateral sclerosis and ageing [20].	Gene expression increased in placental tissue of stillbirth and late-term pregnancy; associated with increased lipid peroxidation in late-term and stillbirth placentae [170].
Xanthine Oxidoreductase	liver, gut, lungs, kidney, breast, placenta [54,171,172]	Cytosol, Peroxisomes [171]	hypoxanthine, xanthine, nitrite [171]	xanthine, uric acid, nitric oxide [145]	O_2_^•−^, H_2_O_2_ [56,169]	Excess uric acid leads to gout, hyperuricemia; deficiency of enzyme leads to excess xanthine and potential renal failure [52,53].	Increased expression and activity of XOR found in placentae (invasive cytotrophoblasts) of pre-eclamptic women [173]. Hyperuricemia associated with pre-eclampsia and GDM [174].
Sulfite Oxidase	liver, kidney, heart, skeletal muscle, brain, placenta [175]	Mitochondria (intermembrane space) [176]	sulfite, nitrite	sulfate, nitric oxide [177]	O_2_^•−^, H_2_O_2_ in plant SO [178,179]	Deficiency leads to toxic sulfite accumulation and brain damage, neurological abnormalities, myoclonic seizures, and neonatal or early-childhood mortality [50,180].	Deficiency leads to toxic sulfite accumulation and brain damage, neurological abnormalities, myoclonic seizures and neonatal or early-childhood mortality following uneventful pregnancies. Brain damage begins in utero [180].
Mitochondrial Amidoxime Component 1 & 2	liver, kidney, adipose tissue [181]	mitochondria (outer mitochondrial membrane), Peroxisomes (mARC2) [182,183]	nitrite; *N*-hydroxylated compounds/prodrugs [183]	nitric oxide; corresponding nucleoside/drug [183,184,185]	Not explored	Implicated in lipid metabolism and fatty liver disease [186], mARC1 deficiency was found to lower blood cholesterol levels and protect against cirrhosis [187].	mARC1 was detected in both adult and foetal livers; mARC2 protein was only present in adult liver [181].

Abbreviations: GDM, gestational diabetes mellitus; H_2_O_2_, hydrogen peroxide; O_2_^•−^, superoxide radical; mARC, mitochondrial amidoxime-reducing component; SO, sulfite oxidase; XOR, xanthine oxidoreductase.

### 4.1. Molybdoenzyme Associations with Redox Status in Pregnancy

The molybdoenzyme families mediate many cellular reactions, simultaneously generating reactive oxygen species, all of which are capable of modulating the maternal/foetal unit. 

#### 4.1.1. Aldehyde Oxidase

Extensive research has been devoted to the drug metabolising properties of AOX, but endogenous substrates are still being sought. AOX is a generator of all-trans retinoic acid through the conversion of retinaldehyde, implicating this enzyme in vitamin A metabolism, which is crucial for the developing foetus, but can be teratogenic in excess [188]. Maiti, Sultana, and colleagues were some of the first to research the potential links between ROS damage, placental dysfunction and molybdoenzyme expression in the development of a pathological state during gestation [170]. They found that the markers of nucleic and lipid oxidation 8OHdG and 4HNE, were significantly increased in late-term and stillbirth placentas in comparison to term, and that there was increased aldehyde oxidase expression in late-term (>41 weeks)-aged placentas [170]. Maiti and colleagues, furthermore, found that inhibition of AOX with raloxifene in placental explants reduced lipid peroxidation, further implicating AOX in the mediation of oxidative damage in the placental tissue. 

#### 4.1.2. Xanthine Oxidoreductase

XOR is unusual in that it exists in two interconvertible forms, xanthine dehydrogenase (XDH) and xanthine oxidase (XO), which differ in their reduction capacity, with XO reducing oxygen only, and XDH reducing both oxygen and NAD+, with a preference for the latter [19,171]. Both forms can catalyse the reaction of hypoxanthine to xanthine, and xanthine to uric acid, making XOR an essential mammalian enzyme for the degradation of purines. However, alongside the reactive oxygen species generated during these conversions, the final product of XOR activity, uric acid, is also a pro-oxidant. While capable of scavenging reactive oxygen species as an antioxidant in a hydrophilic environment, uric acid is overwhelmingly linked to the oxidation of lipid membranes and low-density lipoprotein in hydrophobic environments, generating cellular damage [189]. Concomitant with uric acid production, XO-generated superoxide radicals can furthermore inhibit NO production and promote endothelial dysfunction [190]. High uric acid levels are associated with obesity, insulin resistance, diabetes, and critically, during pregnancy, with pre-eclampsia and GDM [191]. Elevated xanthine levels in maternal plasma was noted in pregnancies complicated by GDM, which was tentatively linked to xanthine oxidase activity in one small (*n* = 143) study [192]. In a Chinese cohort of 85,609 pregnant individuals, elevated uric acid in maternal serum was associated with a significantly increased risk of GDM between 13 and 18 weeks’ gestation [174]. XO activity has been associated with pre-eclampsia and hypoxia in gestational complications. XO has been implicated in postischemic reperfusion injury via the generation of ROS in cytotrophoblasts of pre-eclamptic women [173]. The hyperuricemia observed in preeclampsia, initially solely attributed to impaired renal function, is now also considered a result of increased xanthine oxidase activity, in converting xanthine to uric acid. Oxidative stress generated by xanthine oxidase activity, resulted furthermore in increased apoptosis and decreased invasive and differentiation capability of an immortalised human EVT cell line [193]. 

#### 4.1.3. Sulfite Oxidase 

Sulfite accumulation in neural tissues results in an increase in reactive oxygen species and the depletion of antioxidant defences, including the decreased functioning of crucial enzymes such as glutathione peroxidase, and disrupts mitochondrial biogenesis and function [194]. Thus, sulfite oxidase activity is necessary in mammalian cells and for neurological development. Increased sulfite oxidase activity was hypothesised to confer a therapeutic effect during pregnancy, in a novel paper investigating nausea and vomiting of pregnancy (NVP). Taylor [195], discusses the fact that hydrogen sulfide (H2S), in tandem with nitric oxide, induces angiogenesis and vasodilation, both crucial to placental development and foetal growth. Augmented H2S production during pregnancy may cause increased nausea, and its catabolism to sulfite, leading to sulfite accumulation, is linked to vomiting and feeding difficulties, as sulfite can irritate the gastrointestinal tract [195]. Sulfite oxidase, which is responsible for the oxidation of sulfite to sulfate, may be a beneficial therapeutic agent in this setting.

### 4.2. Mitochondrial Amidoxime-Reducing Component 1 and 2, and Lipogenesis

Only discovered in 2006 [196], mARC is the fourth molybdoenzyme found in humans, and is capable of reducing hydroxylated compounds, mostly *N*-hydroxylated (NHC), which form prodrugs or mutagens. The paralogues mARC1 and mARC2 require protein partners to reduce substrates; in humans, they are the electron transport proteins cytochrome b5 B (CYB5B) and cytochrome b5 reductase (CYB5R). The mARC proteins have been proposed to act as multifunctional enzymes with varying physiological functions dependent on partners, substrates, tissue, and cellular localisation. 

Developmental modulation of the mARC paralogues was evidenced with prominent mARC1 expression in both adult and foetal (first-trimester) liver, while mARC2 was only identified in the human adult [181]. In the same study, mARC2 protein levels were modulated by nutrition status, and expression was decreased in obese patients following a calorie restriction diet [181]. The mARC proteins have been associated with an increase in lipogenesis and the aetiology of diabetes in animal models and human studies, although the precise mechanisms remain unknown. Tejada-Jimenez and colleagues hypothesised that mARC-driven lipogenesis was mediated by its nitric oxide production, which could interact with fatty acids to generate nitro-fatty acids, signalling molecules with antioxidant properties [197]. More recently, missense and nonsense mutations in the mARC1 gene MARC1 were associated with protection from cirrhosis, reduced levels of hepatic fat, and lower levels of total cholesterol and low-density lipoprotein cholesterol [187], while genetically determined increases in the expression of MARC1 adversely affected liver enzymes, liver fat and circulating lipids, as analysed via Mendelian randomisation [186]. Lewis and colleagues, furthermore, found that a knockdown of MARC1 in primary human hepatocytes reduced lipid accumulation and secretion of apolipoprotein B, a constituent of low-density lipoprotein [186]. Therefore, mARC1 is increasingly attractive as a potential target for enzyme inhibition for the mediation of fatty liver disease and obesity—how these systems are modulated during gestation and placentation however, and their influence on gestational complications, are currently unexplored. 

Further research is essential to elucidate the complex and varied roles of molybdoenzymes in gestational and placental health, particularly regarding the secondary functions of the enzymes as generators of the signalling molecule nitric oxide, alongside traditional enzymatic products and reactive oxygen species.

## 5. Conclusions

Molybdenum is a versatile element integral to multicellular eukaryotic life. However, the literature is broadly lacking in large-scale, population-based studies to critically examine molybdenum exposure, supplementation and status during pregnancy. Molybdenum is currently absent from most prenatal vitamin formulations, apparently under the assumption that ordinary dietary intake of molybdenum is sufficient. This may be the case, but, as previously expressed, molybdenum insufficiency, rather than deficiency, may be especially prevalent during the increasingly nutrient-demanding period of gestation, or as a result of changes to diet due to food aversions and gastroparesis-like symptoms. Animal models provide a promising avenue for uncovering molybdenum’s antioxidative, antidiabetic and lipogenic properties, although exact dosages and avenues of intake will require refinement in translation to human studies. 

The data presented in this review acknowledge and outline the dual capacity of molybdenum to be an antioxidant and beneficial supplement, while also, within the action of molybdoenzymes and potentially independently of them, acting as an oxidative stressor with toxic properties. Further study, across a range of gestational timepoints and using numerous biological tissues to adequately analyse molybdenum status, are integral to elucidating this element’s pathophysiological associations. Increased subjects to improve statistical power are vital. Urinary studies in particular may provide confounding results, due to (a) differences in normalisation factors (such as the use of creatinine or specific gravity for adjustment), (b) severe disease states such as diabetes, which can alter urine output and filtration, and (c) whether a higher urine molybdenum content is an expression of heightened molybdenum exposure (acute or chronic), or molybdenum depletion from serum and tissues. Few studies have examined both serum and urine molybdenum content, and intracellular content is not commonly measured. While serum and urine molybdenum outputs may be reflective of molybdenum intake, particularly in healthy adults, this may not be the case in diseased or highly metabolically active states. Umbilical cord and placental tissue may be useful for evaluating the transfer of molybdenum through the maternal–foetal unit. 

Furthermore, it is worthwhile to elucidate whether the potential antidiabetic and antioxidant effects of molybdenum exposure can be induced independently of molybdoenzyme activity, and to further investigate the functions of molybdoenzymes as regulators of oxidative stress within the cell, in the context of their multifunctional chemistry. We currently hypothesise that molybdenum, like selenium, will exhibit U-shaped associations, meaning that there would be a higher risk of gestational complications and adverse outcomes at lower as well as higher exposure levels, as has previously been speculated for the essential micronutrient selenium. The cellular mechanisms of molybdenum storage and transfer, as well as the recommended daily allowances, also require research, to discover the optimal range of molybdenum intake for a healthy gestational period.

## Figures and Tables

**Table 1:** Selected studies on Molybdenum associations with Metabolic Syndromes in non-pregnant cohorts.

Authors	Method of Detection	Population Number	Population Details	Matrix	Country	Results
Ajibola et al., 2014 [128]	AAS	148 subjects (98 diabetes, 50 control)	Diabetes: 65.3% female, control: no details provided; diabetes: 55.92 ± 12.82 years, control: 42.06 ± 7.31 years	Plasma (fasting)	Nigeria	Significantly increased Mo in diabetics in comparison to control.
Flores et al., 2011 [129]	ICP-MS	88 subjects (52 slight-to-moderate diabetes complications, 24 severe diabetes complications, 12 control	Either sex, no further details provided; 52 ± 8 years	24 h urine, fasting serum	Mexico	Urinary Mo was significantly decreased in severe diabetics in comparison to moderate diabetics; Serum Mo was increased in diabetic patients in comparison to control, and significantly increased in severe vs. moderate diabetic subjects.
Li et al., 2021 [127]	ICP-MS	5356 subjects (2678 ‘metabolic syndrome’ diagnosed, 2678 control)	MetS: 55.2% male, 54.5 (10.9) years; control: 55.2% male, 54.8 (10.8) years	Plasma (fasting)	China	Plasma Mo inversely correlated with risk of Metabolic Syndrome, in a dose-response manner.
Menke et al., 2015 [131]	ICP-MS	9447 subjects (1364 diabetes, 8083 control)	Diabetes: 51.7% ± 1.75 female, 58.6 ± 0.54 years, control: 50.5 ± 0.69 female, 45.6 ± 0.30 years	Spot urine	US	Higher quartiles of Mo were associated with greater HOMA of insulin resistance; Mo was positively associated with diabetes prevalence.
Wang et al., 2020 [133]	ICP-MS	1237 subjects, random selection	Female only; diabetes: 50.0 years, control: 49.5 years	Spontaneously voided urine (first morning)	US	No association found between urinary Mo and diabetes.
Wang et al., 2020 [132]	ICP-MS	1262 subjects, random selection	Female only; 49.7 (2.8) years at baseline	Spontaneously voided urine (first morning)	US	Urinary Mo concentration was significantly inversely associated with HOMA of insulin resistance at baseline.
Yang et al., 2023 [130]	ICP-MS	1423 subjects, random selection	56.5% male, 46.9 ± 17.2 years	Spot urine	US	High urinary Mo levels were associated with elevated FPG and HbA1c levels.

Abbreviations: AAS, flame atomic absorption spectrophotometry; FPG, fasting plasma glucose; ICP-MS, inductively coupled plasma mass spectrometry; MetS, metabolic syndrome.

**Table 2:** Selected studies on Molybdenum associations with Metabolic Syndromes in pregnancy.

Authors	Method of Detection	Population Number	Population Details	Matrix	Country	Results
Al-Saleh et al., 2004 [138]	AAS	30 subjects (15 gestational diabetics, 15 control)	Late third trimester (gestational diabetics: 39.0 ± 0.3 weeks, control: 40.0 ± 0.4 weeks)	Blood/Serum	Kuwait	No significant difference found in maternal Mo serum concentrations between control and GDM group. Significantly increased Mo concentrations in umbilical vein and artery of GDM group in comparison to control.
Al-Saleh et al., 2005 [140]	AAS	31 subjects (14 insulin-dependent diabetics, 17 control)	Late third trimester (insulin-dependent diabetics: 38 ± 0.31 weeks, control: 40 ± 0.4 weeks)	Serum	Kuwait	No significant difference in maternal Mo serum concentrations between control and diabetic group. Significantly increased Mo concentrations in umbilical vein and artery of diabetic group in comparison to control.
Al-Saleh et al., 2007 [139]	AAS	21 subjects (10 obese gestational diabetics, 11 control obese)	Late third trimester (obese gestational diabetics: 38.0 ± 0.40 weeks, obese control: 40.0 ± 0.50)	Serum	Kuwait	Increased Mo serum concentration in maternal vein and umbilical artery of obese gestational diabetics in comparison to obese controls.
McAlpine et al., 2019 [137]	ICP-MS	127 subjects (89 used supplements, 38 did not)	Late second and early third trimester, 180–210 days’ gestation (25–30 weeks)	Serum (Fasting)	Australia	No evidence of significant association between Mo in serum or dietary levels and the incidence of GDM.
Zheng et al., 2019 [135]	ICP-MS	1857 healthy non-obese subjects	Late first trimester (median: 12 weeks’ gestation)	Plasma	US	Every 50% increase in molybdenum concentration was associated with 1.2 mg/dL lower mean glucose level (95% CI: −2.3, −0.1 mg/dL)
Zheng et al., 2020 [136]	ICP-MS	1720 healthy non-obese subjects	Late first trimester (median: 12 weeks’ gestation)	Plasma	US	Inverse association between Mo plasma levels and glucose levels, in three different statistical models. Strong associations were not observed in BKMR modelling in comparison to adaptive LASSO and GAM.

Abbreviations: AAS, atomic absorption spectrophotometry; BKMR, Bayesian kernel machine regression; GDM, gestational diabetes mellitus; GAM, generalised adaptive model; ICP-MS, inductively coupled plasma mass spectrometry; LASSO, adaptive least absolute shrinkage and selection operator.

## Data Availability

No new data were created in this study. Data sharing is not applicable to this article.
